# Medical News and Bulletin

**Published:** 1843-11-11

**Authors:** 


### MEDICAL NEWS AND BULLETIN.

### GROSS ON THE WOUNDS OF THE INTESTINES.

We have received the above work, to which we have before called the attention of our readers, and will notice it in our next number.

### CLINICAL LECTURES.

In our next number we shall commence reporting the Clinical Lectures delivered at the several Hospitals and Dispensaries of this City, and continue their publication during the ensuing winter and spring.

### PROFESSOR CHOMEL'S LECTURES.

We shall publish this year two more lectures from Professor Chomel, which will complete the course.

In the first number of the coming year it is our intention to commence a course of Lectures on Fractures, by Professor Velpeau, which have been prepared for this Journal.

### SUCCESSOR OF LARREY IN THE INSTITUTE.

The medical world in Paris had been, for some months before the day of election, in quite a buzz of expectation and *quidnunc-ery* as to who should prove fthe ortunate candidate for the vacant place of the famous Larrey. The distinguished Professor of Montpelier, M. Lallemand, was the favourite, it was generally believed; and this idea was confirmed on finding that the committee, whose part it was to select a certain number of names out of the host of candidates, had placed him at the top of their list. The others followed thus: Lisfranc, Ribes, Velpeau, and Gerdy (equal), Amussat, Begin, and Jobert. The indefatigable surgeon of La Charité, however, although only fourth on the list, proved at last to be the successful competitor. The French Journal, L'Experience, approves highly of the choice, and concludes its notice of the election with these words: "M. Velpeau, in point of professional fame, is one of the reigning prince s of surgery. His works on operative surgery and topographic anatomy are known everywhere. He possesses in the highest degree a love for his art. His intellectual character is eminently progressive; if he errs in this respect, it is rather in the way of *too much* than of *too little.* His whole career has been one of labour and study; and no one has better proved by example the truth of the adage—

'Labor improbus omnia vincit.'"

(This *eloge*, we believe, is nothing more than true.)

M. Lisfranc does not fare so well. Some of the French journals are having a fling at him for a very notable piece of puffery with which he is charged. It seems that the wealthy Marquis d'Aligre recently had the misfortune of breaking the neck of one of his thigh bones, and that M. Lisfranc was called in to attend him. This was all duly announced in the public journals, as a matter of course, at the time of the accident; and the profession naturally thought that there was an end of the matter, at least between the public and the doctor. Not so, however; for we are told that many of the leading fashionables in Paris had the honour of receiving, some weeks after the accident, the following 'jolie petite lettre, fort coquettement lithographée:'—

"The apparatus was yesterday removed from the limb of M. le Marquis d'Aligre. The fracture has healed, without any shortening of the member. From this time the Marquis enters upon convalescence; he will soon be able to walk; his general health is excellent. (Signed)

Lisfranc. *Nauche.*"

*Paris*, 23d February, 1843.

*Medico-Chirurgical Review*, Oct. 1843.

### DECADENCE OF A BRILLIANT OPERATION!

How, in the name of all that is wonderful, comes it that we never hear now of dividing people's tongues—or, as we heard an elocution-professor once tell a public audience, of cutting people's throats—for the cure of stammering? This great achievement of modern surgery has lasted just about as long as a fashionable novel usually does: before a twelvemonth has expired, it is not even talked of. Last year the weekly periodicals teemed with cases of wonderful cures effected "slick off in a twinkling;" and now, alas! there is an utter dearth of any thing that excites curiosity. By mere accident we noticed the heading of an article in a late number of the Annales de la Chirurgie—by-the-bye, one of the best of the French journals—on the treatment of stammering by dividing the genio-glossi, and we were curious enough to see what the author would now say upon the defunct subject. With most praiseworthy candour, he tells his readers that, although he met with very decided success in some cases, the result of the operation in his practice, on the whole, was so unsatisfactory that for the last eight months he had entirely given it up. The editor of the journal, in a foot-note, remarks: "It is this conclusion alone that has induced us to insert the memoir of M. Cloesser; for it is more remarkable for its good faith, than for any importance of its statements.—*Ibid.*

*Compliment to the Memory of Sir Charles Bell.*—Sir Robert Peel has addressed the following letter to lady Bell:—

"Madam,—I have had great pleasure in recommending to her Majesty, that in consideration of the high attainments of your lamented husband, and the services rendered by him to the cause of science, a pension of one hundred pounds per annum for your life, shall be granted you, from that very limited fund which Parliament has placed at the disposal of the Crown, for the reward and encouragement of scientific labours.

This pension, small in amount as it necessarily is, will perhaps be acceptable to you, as a public acknowledgement, on the part of the Crown, of the distinguished merit of Sir Charles Bell.

I have the honour to be, madam, your faithful and obedient servant.

Robert Peel."

